# Comparison between harvesting and preserving the spinous process for adolescent idiopathic scoliosis

**DOI:** 10.1186/s12891-016-1222-5

**Published:** 2016-08-24

**Authors:** Yu-Cheng Yeh, Chi-Chien Niu, Lih-Huei Chen, Wen-Jer Chen, Po-Liang Lai

**Affiliations:** 1 Department of Orthopaedic Surgery, Chang Gung Memorial Hospital at Linkou, No. 5, Fuxing St., Guishan Dist., Taoyuan City, 33305 Taiwan; 2 Bone and Joint Research Center, Chang Gung Memorial Hospital at Linkou, Taoyuan, Taiwan; 3 College of Medicine, Chang Gung University, Taoyuan, Taiwan

**Keywords:** Adolescent idiopathic scoliosis, Local autologous bone graft, Pedicle screw instrumentations, Posterior fusion, Pseudoarthrosis, Spinous process

## Abstract

**Background:**

Spinous process has been routinely resected during posterior fusion of adolescent idiopathic scoliosis for fusion bed preparation and local autologous bone graft supplement. However, spinous process serves as an important structure in posterior ligament complex and was the anchorage of paraspinal muscle groups. With the development of pedicle screws instrumentation and the potential fusion ability in children, the need for resecting spinous process in this procedure could be further investigated. The purpose of this study was to compare the fusion rates, surgical outcomes and complications between harvesting and preserving the spinous process in posterior fusion of adolescent idiopathic scoliosis.

**Methods:**

From January 2003 to December 2008, 104 consecutive adolescent idiopathic scoliosis patients underwent primary posterior fusion with local autologous bone grafts and following for a minimum of 24 months were reviewed. The patients were divided into a harvesting group (*n* = 61) with the spinous process harvested, and a preserving group (*n* = 43) with the spinous process preserved. Blood loss, radiographic assessments, and clinical outcomes were compared between the two groups.

**Results:**

There were no significant differences in duration of surgery and peri-operative blood transfusion between the two groups. However, blood loss was statistically greater (983 ± 446 ml vs. 824 ± 361 ml; *p* = 0.048) and duration of hospitalization was statistically longer (7.4 ± 1.0 days vs. 6.8 ± 0.8 days; *p* = 0.003) in the harvesting group. The pre- and post-operative structural curves, correction rates, sagittal profile and loss of corrections were similar in both groups. Based on radiographic evaluation, the incidences of pseudoarthrosis were similar in both groups (3/61 vs. 2/43; *p* = 0.95). The incidence of prescribing pain medication for back discomfort during follow-up was statistically higher in the harvesting group (16/61 vs. 4/43; *p* = 0.03).

**Conclusions:**

The surgical outcomes and fusion rates between harvesting and preserving the spinous process were comparable. Resecting the spinous process as local autologous bone graft may not be necessary in posterior fusion for adolescent idiopathic scoliosis patients.

## Background

Spinal arthrodesis with instrumentation and bone grafting is the mainstay of surgical treatment for adolescent idiopathic scoliosis (AIS). Although autologous iliac crest bone graft was once considered to be the gold standard since it has osteogenic, osteoconductive and osteoinductive properties, donor-site morbidities including hematoma, infection, donor-site pain and sensory deficits have been frequently reported [1–3]. Therefore, alternative sources of bone graft such as allograft and various biomaterials have been applied with comparable results [4–6].

Local autologous bone graft has been recommended for the optimal surgical care of AIS surgery and could avoid the donor site comorbidities from harvesting iliac crest bone graft [7, 8]. To expand the volume of bone graft material, artificial bone graft substitutes such as calcium sulfate or calcium phosphate can also be used in combination with local autologous bone graft [9–12].

Bone chips obtained from spinous process resection, lamina decortication and facetectomy are typically used as local autologous bone graft in posterior spinal fusion surgery [13]. However, spinous process resection causes damage to the integrity of the posterior ligament complex, including the spinous process, and supraspinous and interspinous ligaments, which may lead to adjacent spinal instability [14]. Furthermore, resecting the spinous process would also sacrifice the anchorage of paraspinal muscles. Whether removing the spinous process to harvest more local autologous bone graft would have a positive impact on the outcomes of AIS surgery is unknown. The purpose of this study was to compare the duration of surgery, blood loss, surgical outcomes and complication rates between preserving and harvesting the spinous process for the posterior fusion of AIS.

## Methods

### Patients

This was a retrospective study analyzing a consecutive series of patients with AIS who received posterior fusion and instrumentation at our institute. This study was approved by the Institute Review Board (CGMH 104-1404B) of our hospital. All of the patients underwent surgery between January 2003 and December 2008. The patients included in this study were 1) aged from 10 to 19 years old, 2) received primary posterior fusion and instrumentation, 3) used local autologous bone chips with the supplementation of calcium sulfate, and 4) were followed up until bone maturity or a minimum of 24 months.

These patients were categorized into two groups: a harvesting group who had their spinous process harvested, and a preserving group who had their spinous process preserved. The decision to harvest or preserve the spinous process for each patient was based on the attending physicians’ preference. However, all of the patients were operated by the same surgical team which included two attending physicians and one rotating resident. Both groups of patients received partial facetectomy and lamina decortication. All scoliotic curves were classified and operated according to the guidelines described by Lenke [15].

### Surgical techniques

A standard midline incision was made first with the patient in a prone position. The soft tissue was then carefully dissected until full exposure was achieved from the upper tip of the cephalad vertebrae to the inferior facet of the caudal vertebrae and laterally to obtain full exposure of the facet joint. After pedicle screws had been placed, a portable X-ray device was used to check the pedicle screw position. Rods were then inserted and the curves were corrected by derotation of the rods with cantilever bending if necessary.

**Fig. 1:**
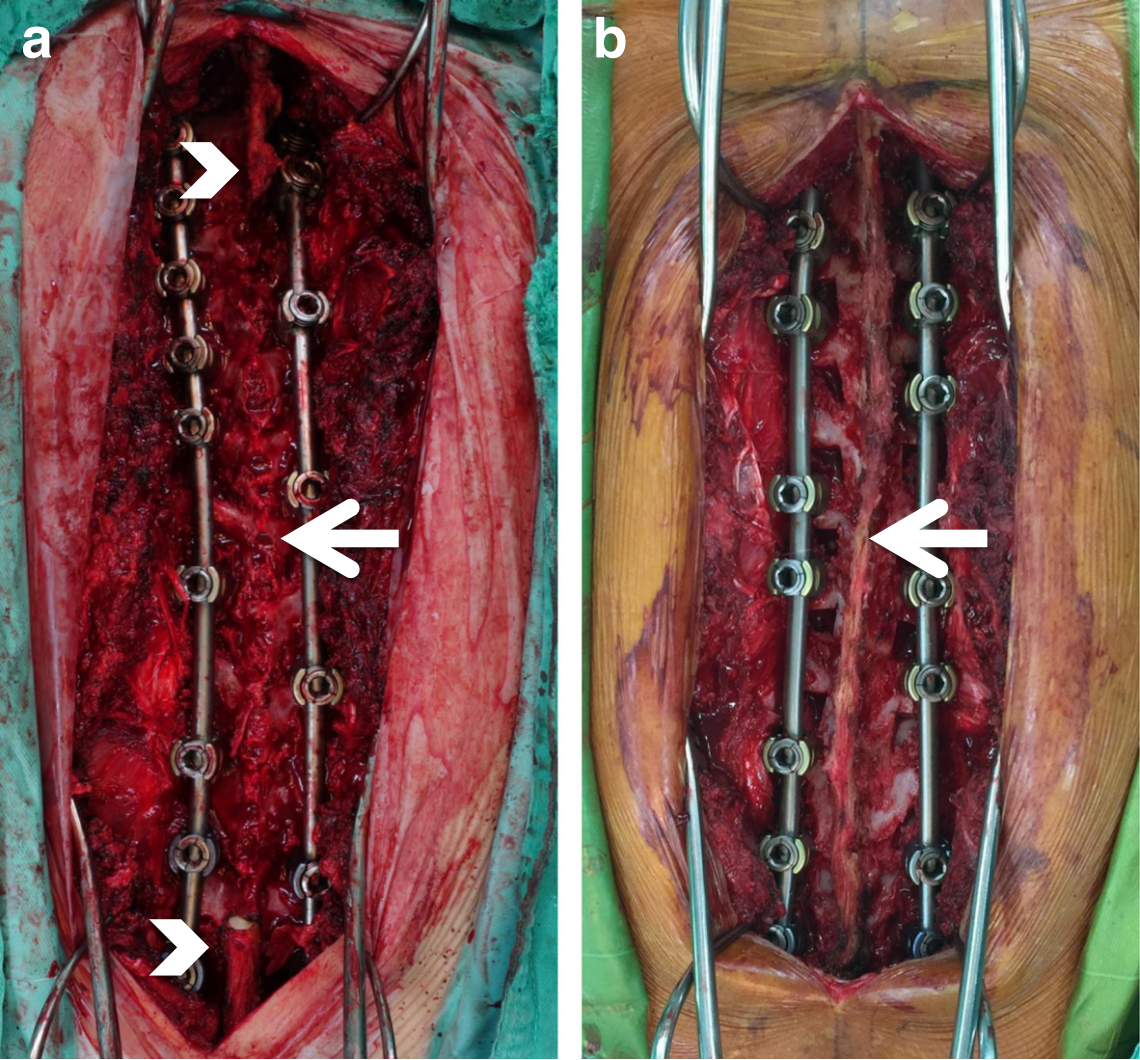
**a** Harvesting group: Resecting the spinous process (*arrow*) as additional local bone graft. Note: The most cephalad and caudal vertebra (*arrow head*) were spared to avoid jeopardizing the posterior complex of un-fused segments. **b** Preserving group: Preserving the spinous process, supraspinous ligament and interspinous ligament (*arrow*)

The bone chips from lamina decortication and partial facetectomy of the fused vertebra were used as autologous bone graft in both the harvesting and the preserving groups. In the harvesting group, the spinous processes of the fused vertebra were resected and morselized as additional bone chips (Fig. 1). Nevertheless, the spinous processes of the uppermost and lowest fused vertebrae were spared to avoid jeopardizing the posterior complex of un-fused segments. In both groups, 20 g of commercially available calcium sulfate was mixed with the morselized bone chips and then gently placed on the previously decorticated surface of the facets and laminae with full coverage of the fusion segments.

Electroneurophysiological monitoring was performed during the operation. The wounds were closed in layers with the placement of a drainage tube. Standing and walking were encouraged on the second post-operative day with the application of thoracolumbar orthosis.

### Assessment of results

The medical records of all patients were collected under the approval of the Institutional Review Board of our hospital. The intraoperative blood loss, duration of surgery, amount of total inserted implants, total peri-operative blood transfusion and duration of hospitalization were recorded. There was no additional medication given peri-operatively to reduce blood loss in all patients. The differences between the two groups were tested using the independent *t*-test. A *p* value of less than 0.05 was considered to be statistically significant in this study.

The curve patterns were classified according to the Lenke classification [15]. Whole spinal column anteroposterior and lateral images were taken and reviewed pre-operatively, 3 days, and 3 and 6 months post-operatively, and then annually. Data of all pre- and post-operative structural curves were collected to compare the surgical outcomes. Pre-operative and post-operative sagittal profile including thoracic kyphosis (T5-T12) and lumbar lordosis (L1-S1) were also recorded. Differences in post-operative Cobb angle correction and loss of correction at the last follow-up visit between the two groups were tested using the independent *t*-test.

Pseudoarthrosis was determined by static radiographic findings, including implant failure, implant breakage and screw halo sign. In addition, the pain medications prescribed for post-operative back pain or soreness during follow-up was specifically recorded. The incidence differences between the two groups were tested using the chi-square test. Other complications related to the surgery, including wound infection, hematoma, wound dehiscence, and the need for revision surgery were also recorded and analyzed.

## Results

One hundred and four patients (11 males and 93 females) met the inclusion criteria and were analyzed (Table 1). There were 61 patients (10 males and 51 females) in the harvesting group with an average age of 14.0 years (range: 11 to 19 years). There were 43 patients (one male and 42 females) in the preserving group with an average age of 13.8 years (range: 10 to 18 years).

**Table 1:** Patient demographics and peri-operative variables

	Group I (Harvesting)	Group II (Preserving)	*p* value*
Patients	61 (10 M & 51 F)	43 (1 M & 42 F)	
Age (years)	14.0 (11 ~ 19)	13.8 (10 ~ 18)	0.71
Median f/u length (months)	60 (24 ~ 128)	73 (24 ~ 107)	
Level fused	10.6 ± 1.7	10.0 ± 1.9	0.06
Duration of surgery (minutes)	216 ± 47	224 ± 50	0.40
Implants inserted (numbers)	13.3 ± 2.2	13.3 ± 2.0	0.97
Blood loss (ml)	983 ± 446	824 ± 361	0.048
Peri-OP blood transfusion (units)	6.0 ± 3.3	5.1 ± 3.0	0.13
Hospitalization length (days)	7.4 ± 1.0	6.8 ± 0.8	0.003

Data are presented as mean ± standard deviation (SD)

*M* male, *F* female, *OP* operative, *f/u* follow-up

*p value was calculated by unpaired two-sample *t*-test

The number of patients categorized according to Lenke classification as Lenke 1, 2, 3, 4, 5 and 6 were 33 (54 %), 12 (20 %), 7 (11 %), 1 (2 %), 5 (8 %) and 3 (5 %) in the harvesting group and 24 (56 %), 7 (16 %), 1 (2 %), 0 (0 %), 8 (19 %) and 3 (7 %) in the preserving group, respectively. The median follow-up duration was 60 months (range: from 24 to 128 months, average 62 months) in the harvesting group and 73 months (range: from 24 to 107 months, average 72 months) in the preserving group. The average fused level was 10.6 ± 1.7 (range: from 6 to 14) in the harvesting group and 10.0 ± 1.9 (range from: 7 to 14) in the preserving group, with no statistical difference (*p* = 0.06).

The average durations of surgery were 216 ± 47 min in the harvesting group and 224 ± 50 min in the preserving group, with no statistical difference (*p* = 0.40). However, the blood loss in the harvesting group (average 983 ± 446 ml) was statistically greater than in the preserving group (average 824 ± 361 ml; *p* = 0.048). The average amounts of total inserted implants were 13.3 ± 2.2 in the harvesting group and 13.3 ± 2.0 in the preserving group (*p* = 0.97). The average peri-operative blood transfusions were 6.0 ± 3.3 units in the harvesting group and 5.1 ± 3.0 units in the preserving group, with no statistical difference (*p* = 0.13). The duration of hospitalization in the harvesting group (average 7.4 ± 1.0 days) was slightly longer than in the preserving group (average 6.8 ± 0.8 days; *p* = 0.003).

Table 2 shows the radiographic measurements. The average pre-operative structural curves were 50.7° (range: 34 to 82°) in the harvesting group and 51.3° (range: 27 to 78°) in the preserving group (*p* = 0.74). The average post-operative structural curves were 18.3° (range: 0 to 32°) in the harvesting group and 17.2° (range: 0 to 36°) in the preserving group (*p* = 0.41). The average post-operative structural curve correction rates were 63.3 % (range: 22.2 to 100 %) in the harvesting group and 65.2 % (range: 11.4 to 100 %) in the preserving group (*p* = 0.50). For sagittal profile, the average pre-operative thoracic kyphosis were 20.9° (range: 0 to 60°) in the harvesting group and 21.3° (range: 0 to 63°) in the preserving group (*p* = 0.80). The average post-operative thoracic kyphosis were 24.8° (range: 4 to 57°) in the harvesting group and 25.1° (range: 3 to 60°) in the preserving group (*p* = 0.73). The average pre-operative lumbar lordosis were 54.0° (range: 20 to 79°) in the harvesting group and 54.1° (range: 17 to 83°) in the preserving group (*p* = 0.89). The post-operative lumbar lordosis were 56.8° (range: 15 to 87°) in the harvesting group and 56.2° (range: 13 to 89°) in the preserving group (*p* = 0.68). With regards to major curve correction, the average pre-operative major curves were 53.5° (range: 40 to 82°) in the harvesting group and 54.0° (range: 40 to 78°) in the preserving group (*p* = 0.82). The average post-operative major curve correction rates were 66.0 % (range: 50 to 100 %) in the harvesting group and 70.4 % (range: 52.6 to 100 %) in the preserving group (*p* = 0.50). The average structural curve losses of correction at the last follow-up were 1.8° (range: 0 to 18°) in the harvesting group and 2.7° (range: 0 to 11°) in the preserving group (*p* = 0.25). There were no significant differences in terms of pre- and post-operative curves, correction rates and loss of correction at the last follow-up.

**Table 2:** Radiographic findings

	Group I (Harvesting)	Group II (Preserving)	*p* value*
Structural curve^a^	85	54	
Pre-OP structural curve	50.7° ± 9.5°	51.3° ± 10.8°	0.74
Post-OP structural curve	18.3° ± 6.4°	17.2° ± 8.2°	0.41
Structural curve correction rate	63.3 % ± 13.4 %	65.2 % ± 18.5 %	0.50
Pre-OP thoracic kyphosis (T5-T12)	20.9° ± 11.3°	21.3° ± 11.6°	0.80
Post-OP thoracic kyphosis (T5-T12)	24.8° ± 10.2°	25.1° ± 10.4°	0.73
Pre-OP lumbar lordosis (L1-S1)	54.0° ± 11.5°	54.1° ± 11.7°	0.89
Post-OP lumbar lordosis (L1-S1)	56.8° ± 12.2°	56.2° ± 12.1°	0.68
Pre-OP major curve^a^	53.5° ± 9.4°	54.0° ± 8.7°	0.82
Major curve correction rate	66.0 % ± 9.7 %	70.4 % ± 12.9 %	0.06
Structural curve loss of correction	1.8°	2.7°	0.25

Data are presented as mean ± standard deviation (SD)

*OP* operative

*p value was calculated by unpaired two-sample *t*-test

^a^Structural curve and major curve were defined by Lenke classification

Three of 61 patients (5 %) in the harvesting group and two of 43 patients (5 %) in the preserving group had pseudoarthrosis (*p* = 0.95) based on the radiographic findings (Table 3). In the harvesting group, one patient developed bilateral L4 screw loosening 24 months post-operatively. No clinical symptoms were reported and the structural curve progression was less than 5°. The other two cases were one right T3 screw nut dislodgement and one left L3 screw breakage at post-operative 3 months and 19 months, respectively. Both of the two cases had more than 10° of structural curves progression. While in the preserving group, one patient developed right L1 screw breakage 50 months post-operatively and the other had left L4 screw cap loosening 27 months post-operatively. Less than 5° loss of correction and no complaints of back pain or soreness were reported in these two patients. There was one infection case in the harvesting group at post-operative 6 months. Recurrent infection developed despite serial debridement, so the implant was removed 14 months after the primary surgery. The recorded incidences of prescribing pain medications for post-operative back pain or soreness during follow-up in the harvesting and the preserving groups were 16/61 (26 %) and 4/43 (9 %), respectively (*p* = 0.03).

**Table 3:** Adverse results

	Group I (Harvesting)	Group II (Preserving)	*p* value*
Pseudoarthrosis	3/61 (5 %)	2/43 (5 %)	0.95
Infection	1	0	
Prescribed pain medication	16/61 (26 %)	4/43 (9 %)	0.03

*p value was calculated by chi-square test

## Discussion

Local autologous bone grafting in posterior spinal fusion surgery requires no additional surgical incision and thus avoids most of the morbidities associated with iliac crest bone grafts. In 2004, Violas et al. [7] first proposed using only local autologous bone graft in AIS posterior fusion with pedicle screw instrumentation and reported good outcomes. In addition, Yang et al. [16] reported the combination of local autologous bone graft and allograft yielded no failure of fusion in AIS patients receiving posterior fusion with pedicle screw instrumentation. In AIS surgery, local autologous bone graft has been shown to achieve comparable fusion results to iliac crest bone graft in the past 10 years, and has been recommended for the optimal surgical care of patients undergoing AIS [8]. Local autologous bone graft combined with calcium sulfate has also been shown to be an acceptable alternative for iliac crest autograft in single or multiple level posterolateral spinal fusion [10–12].

The spinous process is typically harvested during posterior spinal fusion in order to obtain enough graft material to facilitate fusion [13], and the amount of local autologous bone graft harvested may play an important role in successful fusion. In our study, the harvested bone chips per fusion level contained one spinous process and two facetectomy bone chips in the harvesting group, and two facetectomy bone chips in the preserving group. In general, the relative amounts of bone chips were proportional to the fusion levels. Although the absolute amounts of local autologous bone chips used in both groups were not specifically measured during surgery, facetectomy alone with calcium sulfate supplement (the preserving group) still achieved comparable fusion results as the harvesting group. All of the patients maintained good reduction with a low loss of correction rate post-operatively.

Pseudoathrosis was determined by plain radiographic findings such as implant failure or screw halo signs in our study. Although computed tomography (CT) for post-operative fusion status evaluation is more sensitive than plain radiographs, it can lead to excessive radiation exposure in adolescent patients. In our study, the preserving group, who had their spinous process preserved, had a comparably low pseudoarthrosis rate as the harvesting group, who had their spinous process resected. Among the five patients with pseudoarthrosis, only two cases in the harvesting group developed curve progression of more than 10°, whereas other three cases maintained curve progression within 5°.

A high spontaneous fusion rate in early onset scoliosis patients undergoing growth rod exchange surgery has been well documented [17–19]. Extensive extraperiosteal dissection and prolonged immobilization of the instruments have been reported to be risk factors for spontaneous fusion after spinal surgery [19, 20]. Furthermore, pedicle screw-based instrumentation could provide rigid fixation for all three spinal columns, and has shown improved coronal curve correction and satisfaction in AIS patients [21–23]. Although AIS patients do not have the same spontaneous fusion ability as those with early onset scoliosis, the potential fusion ability in a skeletally immature spine and rigid fixation of pedicle screw instrumentation may explain why the preserving group achieved a comparably low pseudoarthrosis rate as the harvesting group.

In this study, the surgical blood loss in the harvesting group was statistically greater than in the preserving group (*p* = 0.048). Resecting the spinous process harvested more local autologous bone chips but exposed more cancellous bony surfaces during preparation of the fusion surface. Given the comparable amount of total inserted implants in both the harvesting and the preserving groups (*p* = 0.97), the blood loss from implants implantation could be considered similar. Therefore, the extra exposure of cancellous bony surfaces and the subsequent bleeding caused by resecting spinous process may be the reason for statistically greater blood loss in the harvesting group. In the other hand, although the peri-operative blood transfusion didn’t demonstrate the same statistical differences as the surgical blood loss, there was a trend of less blood transfused in the preserving group (6.0 ± 3.3 units vs. 5.1 ± 3.0 units; *p* = 0.13).

Resecting the spinous process causes damage to the integrity of the posterior spinal complex, including the spinous process, supraspinous ligament and interspinous ligament, which may lead to adjacent spinal instability and failure of the posterior tension band mechanism [14]. The posterior complex sustains most tensile force during flexion of the vertebral column, which is the most pronounced movement requiring anterior compression of the intervertebral discs and gliding separation of the articular facets. Furthermore, resecting the spinous process sacrifices the anchorage of paraspinal muscle groups. Preserving the spinous process and its attached musculoligamentous structures has been reported to result in less post-operative back pain after lumbar decompression or fusion surgery [24, 25]. In this study, the harvesting group had shown slightly longer hospitalization duration (7.4 ± 1.0 days vs 6.8 ± 0.8 days; *p* = 0.003) and had higher incidence of prescribing pain medications for back pain or soreness during the follow-up visits (26 % vs. 9 %, *p* = 0.03). All back pain or soreness was minor within short-term post-operative period, and subsequently resolved after oral medication.

The main limitations of this study included its retrospective nature, lack of statistical pain scores and patient-based outcome evaluations. Although statistical tendencies of less blood loss, shorter hospitalization and lower incidence of prescribing pain medication were observed in patients who had their spinous process preserved, further prospective studies and long term surgical outcome comparison can provide stronger evidence.

## Conclusions

In summary, the surgical outcomes and fusion rates between harvesting and preserving the spinous process were comparable in this study. With the rigid fixation of pedicle screw instrumentation and artificial bone substitute as supplementation for fusion materials, resecting the spinous process as local autologous bone graft may not be necessary in posterior fusion for adolescent idiopathic scoliosis.

## Abbreviation

AIS: Adolescent idiopathic scoliosis
